# Factors influencing the effect of external cephalic version: a retrospective nationwide cohort analysis

**DOI:** 10.1007/s00404-022-06763-2

**Published:** 2022-09-07

**Authors:** Ambrogio P. Londero, Anjeza Xholli, Claudia Massarotti, Arrigo Fruscalzo, Angelo Cagnacci

**Affiliations:** 1 Academic Unit of Obstetrics and Gynaecology, Department of Neuroscience, Rehabilitation, Ophthalmology, Genetics, Maternal and Infant Health, University of Genoa, IRCCS-Policlinico San Martino, Largo Rosanna Benzi, 10, 16132 Genoa, GE Italy; 2 Clinic of Obstetrics and Gynecology, University Hospital of Fribourg, Fribourg, Switzerland

**Keywords:** External cephalic version, Breech, Female, Male, Cesarean section, Operative delivery

## Abstract

**Objective:**

This study aims to assess the factors associated with the success and failure rate of the external cephalic version (ECV) in breech fetuses. Secondary outcomes were fetal presentation in labor and mode of delivery.

**Methods:**

This cross-sectional study examined the live birth certificates from 2003 through 2020 from US states and territories that implemented the 2003 revision. A total of 149,671 singleton pregnancies with information about ECV success or failure were included. The outcome was ECV success/failure, while the exposures were possible factors associated with the outcome.

**Results:**

The successful ECV procedures were 96,137 (64.23%). Among the successful ECV procedures, the prevalence of spontaneous vaginal delivery was 71.63%. Among the failed ECV procedures, 24.74% had a cephalic presentation at delivery, but 63.11% of these pregnancies were delivered by cesarean section. Nulliparity, female sex, low fetal weight centile, high pre-pregnancy BMI, high BMI at delivery, and high maternal weight gain during pregnancy were associated with an increased ECV failure (*p* < 0.001). African American, American Indian and Alaska Native race categories were significant protective factors against ECV failure (*p* < 0.001). Maternal age had a U-shape risk profile, whereas younger maternal age (< 25 years) and old maternal age (> 40 years) were significant protective factors against ECV failure (*p* < 0.001).

**Conclusions:**

A high prevalence of successful ECV procedures and subsequent spontaneous vaginal delivery were found. The present results found nulliparity, maternal race, maternal age, female fetal sex, low fetal weight, and maternal anthropometric features correlated to ECV results. These findings can potentially improve the knowledge about the factors involved in ECV, allowing more informed counseling to the women undergoing this procedure.

**Supplementary Information:**

The online version contains supplementary material available at 10.1007/s00404-022-06763-2.

## What does this study add to the clinical work

**Table:** 

This study sought to evaluate possible factors associated with the success/failure of the external cephalic version (ECV) in breech fetuses. Nulliparity, female fetal sex, low fetal weight, high pre-pregnancy BMI, high BMI at delivery, and increased weight gain during pregnancy, were negatively correlated to ECV success. Black, American Indian and Alaska Native race categories were associated with ECV success. A U-shape relation to risk was found for maternal age, showing an increase in ECV success at the extremes of the curve. These results can improve the knowledge about the ECV-associated factors, allowing better-informed counseling for women undergoing this procedure. Highlights: Nulliparity, female fetal sex, low fetal weight, high pre-pregnancy BMI, high BMI at delivery, and increased maternal weight gain during pregnancy were associated with an increased risk of external cephalic version failure; Black, American Indian and Alaska Native race categories were significantly associated with external cephalic version success; A U-shape relation to risk was found for maternal age, showing an increase in external cephalic version success at the extremes of the curve in young and old maternal ages.	

## Introduction

The reduction of cesarean section (CS) deliveries is within the top priorities in the actual agenda of modern obstetrics. Since the increased number of primary CS leads to an increase in maternal morbidity and mortality [1, 2]. Due to the evidence that emerged during the end of the past century and the beginning of the present century, the preferred method of delivery for term breech presentation has been via CS because of the increased fetal morbidity associated with vaginal delivery [3–5]. With the intent to reduce the primary CS rate, the guidelines of the major obstetrics societies recommend the external cephalic version (ECV) of the fetus presenting in breech presentation [6, 7].

The increased burden of fetal complications associated with breech presentation and the women’s decision-making process for the delivery can raise psychological stress and anxiety [8]. For women with a substantial willingness for vaginal delivery, it could be challenging to choose between the diverse options for dealing with breech presentation. Vaginal breech delivery is associated with increased perinatal risks [9]. CS reduces the fetal risk but raises the threat to the mother and future pregnancies [1, 2]. The external cephalic version, if successful, allows a vaginal cephalic delivery but can be associated with complications and failure. A better understanding of the factors that lead to the success or failure of the ECV procedure can lead to improved management and an improved women’s decision-making process. Furthermore, a better understanding of the delivery mode outcome can add valuable information for enhancing these pathways.

The present study aims to assess the factors associated with the success rate of the external cephalic version in breech fetuses. The secondary outcomes were to assess subsequent fetal presentation in labor and the mode of delivery.

## Methods

### Design, setting, and sample

**Fig. 1:**
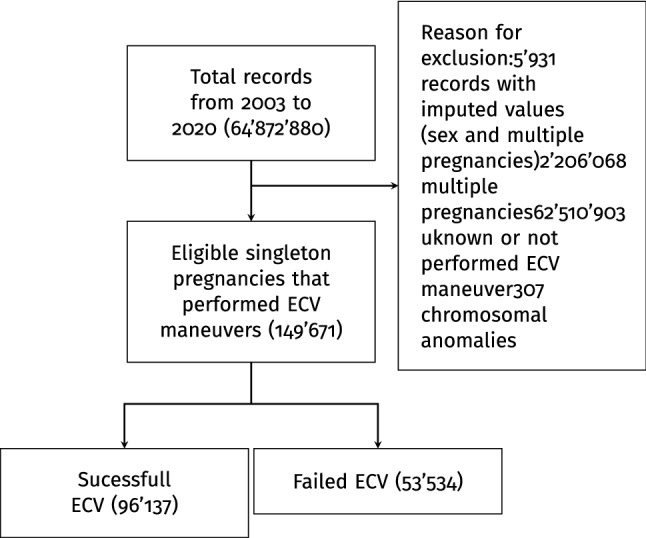
Flowchart of sample selection

This cross-sectional retrospective study employed birth certificate data from the US National Center for Health Statistics as part of the National Vital Statistics System [10]. The period assessed in this study is from 2003 to 2020. Data about ECV success or failure were collected by introducing the 2003 revision of the US Standard Certificate of Live Birth. The new version was phased in and had not full coverage to all States until 2016 [11]. This study used data from 149,671 singleton births undergone a successful or failed external cephalic version in the states that implemented 2003 birth certificate revision and that have recorded the specific data about the considered maneuver. The following inclusion and exclusion criteria were applied during the sampling procedure. All consecutive records of singleton pregnancies reporting information about a successful or failed external cephalic version were included. The following exclusion criteria were applied in succession: records with imputed values for sex or multiple pregnancies, multiple pregnancies, unknown or not performed ECV maneuver, and chromosomal anomalies. Figure 1 shows the flowchart for the population selection. In reporting this study, we have followed the Strengthening the Reporting of Observational Studies in Epidemiology (STROBE) guidelines (http://www.strobe-statement.org/) to ensure that it is documented as thoroughly and accurately as possible. The local Ethics Committee Approval for this study was not required because the data used are de-identified and publicly available. The study was carried out according to the Helsinki declaration.

### Measurement

From the original data sets, the following variables were extracted: maternal age, parity, race, pre-pregnancy body mass index (BMI), BMI at delivery, weight gain during pregnancy, data about successful or failed ECV, fetal presentation at delivery, mode of delivery, multiple pregnancies, multiple pregnancies imputation label, neonatal sex, neonatal sex imputation label, gestational age at delivery, neonatal weight, chromosomal anomalies. A detailed explanation of the variables is available at the following link: https://www.cdc.gov/nchs/data_access/Vitalstatsonline.htm#Tools. Maternal age was stratified into five age classes. Parity was coded as nulliparous vs. parous women. The race was coded according to six categories: single Caucasian race, single African American race, American Indian and Alaska Native (AIAN) single race, Asian single race, Native Hawaiian and Other Pacific Islanders (NHOPI) single race, and more than one race. Neonatal weight centile was calculated using the Hadlock prenatal formula and employing neonatal weight and gestational age at delivery [12]. Then, the Neonatal weight centile was categorized into the following strata: < 10th centile, 10–49th centile, 50–90th centile, and > 90 centiles. Fetal presentation at delivery was classified as Cephalic, Breech, other presentation, and Unknown. Mode of delivery was classified as Spontaneous, Forceps, Vacuum, Cesarean, and Unknown. For all the other variables, unknown values were considered as NA. The previous variables were selected according to the known risk factors for ECV failure or to the favoring factors for ECV success [13–23].

### Data analysis

All analyses were performed using R software (version 4.2.0) [24]. The two-sided probability value *p* < 0.05 was considered statistically significant. The normal distribution of continuous variables was evaluated with the Kolmogorov–Smirnov test. Data are presented as the median and interquartile range (IQR) for continuous non-parametric variables; mean ± standard deviation for continuous parametric variables. Categorical variables (dichotomous or polychotomous) were coded as the percentage and the absolute values, except for NA’s missing values (unless otherwise specified). The results of logistic regression models were presented as odds ratio (OR) and 95% confidence interval (CI.95). The following statistical tests were applied for categorical variables: chi-square test or Fisher exact test. For continuous non-parametric variables, we applied the Wilcoxon test, and for parametric ones, the t-test.

Logistic regression analysis was also performed, considering failed ECV as the dependent variable and possible predictors as an independent variable. We took all possible predictive factors with *p* < 0.05 from univariate analysis in the multivariate model. The multivariate models with and without interactions were analyzed. When considering interactions, the initial multivariate model incorporated all variables and their interactions. When interactions turned out to be non-significant, the analysis without interaction model was employed. In addition, we considered logistic regression models without imputation and with the random imputation of missing values. A sensitivity analysis of the multivariate models was also performed. Pre-pregnancy BMI and BMI at the time of delivery were considered in two separate models because of the strong interaction between the two variables and the higher number of missing values in the BMI at the time of delivery than in pre-pregnancy BMI. The missing values of pre-pregnancy BMI were 26.63%, of BMI at delivery were 61.99%, and of weight gain during pregnancy were 62.44%. The weight gain during pregnancy was not considered in the multivariate analysis because of the high rate of missing values and the strong interaction with BMI. *p* values in multivariate models were adjusted using the false discovery rate test.

## Results

**Fig. 2:**
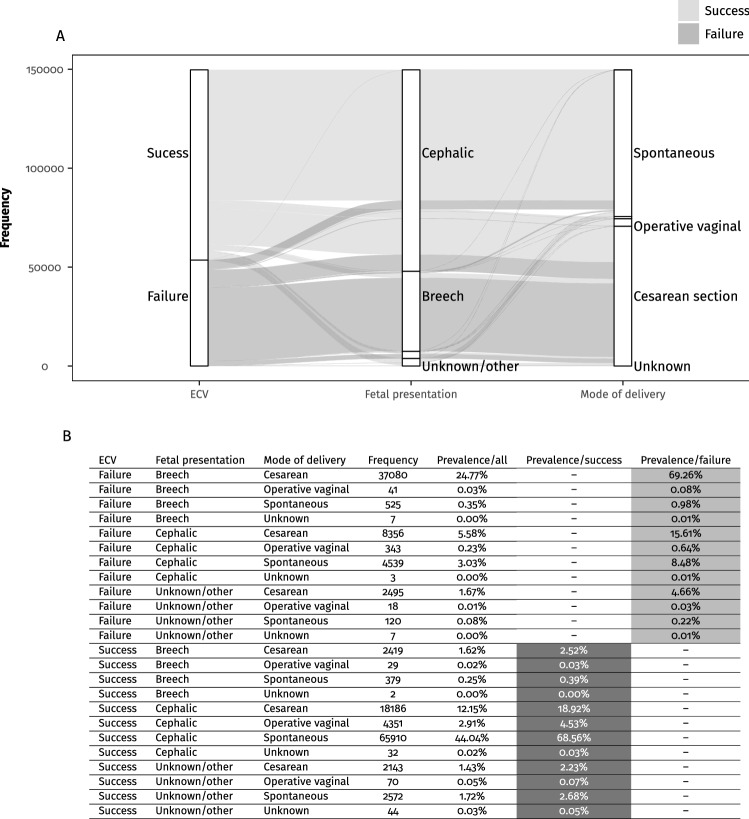
Alluvial plot showing successful or failed ECV following fetal presentation at delivery and delivery modality. **A** Alluvial plot. **B** The prevalence shown in the Alluvial plot

The final study cohort comprises 96,137 successful ECV (64.23%) procedures and 53,534 unsuccessful procedures. The prevalence of female fetuses was 51.34% (76843/149671). Table 1 shows other characteristics of the population studied. Figure 2 shows the pregnancy outcome at delivery considering the successful or unsuccessful ECV. Most of the successful ECVs had a spontaneous birth of a cephalic fetus (Fig. 2A and B). Among the failed ECV procedures, 24.74% had a cephalic presentation at delivery (Fig. 2A and B). 63.11% of these pregnancies will deliver by CS, 34.28% spontaneously, and 2.59% by operative vaginal delivery (the remaining were unknown). Among the failed procedures, 1.06% delivered in breech presentation (spontaneous or operative) (Fig. 2A and B). Among the successful ECV procedures, 2.94% had a breech presentation at delivery, 85.51% of these delivered by CS, 14.43% by vaginal delivery, and the others are unknown (Fig. 2A and B).

**Table 1:** Characteristics of the studied cohort and differences between failed and successful ECV

	All (149,671)	Failed ECV (53,534)	Successful ECV (96,137)	*p*(*)
Maternal characteristics				
Maternal age (years)				
< 20 years	6.79% (10,162/149671)	5.27% (2819/53534)	7.64% (7343/96137)	< 0.001
20–24 years	19.99% (29,919/149671)	18.01% (9642/53534)	21.09% (20,277/96137)	< 0.001
25–29 years	27.82% (41,634/149671)	28.13% (15,059/53534)	27.64% (26,575/96137)	0.044
30–34 years	27.5% (41,166/149671)	30.10% (16,113/53534)	26.06% (25,053/96137)	< 0.001
35–39 years	14.38% (21,528/149671)	15.05% (8057/53534)	14.01% (13,471/96137)	< 0.001
40–44 years	3.28% (4904/149671)	3.21% (1719/53534)	3.31% (3185/96137)	0.288
≥ 45 years	0.24% (358/149671)	0.23% (125/53534)	0.24% (233/96137)	0.736
Pre-pregnancy BMI (kg/m^2^)	25 (21.8–29.8)	25.00 (21.80–30.00)	24.90 (21.80–29.60)	< 0.001
Weight gain (kg)	13.15 (9.07–17.24)	13.61 (9.53–17.69)	13.15 (9.07–17.24)	< 0.001
BMI at delivery (kg/m^2^)	30.47 (27.12–35.02)	30.62 (27.22–35.35)	30.38 (26.99–34.77)	< 0.001
Nulliparity	33.75% (50,510/149671)	41.49% (22,213/53534)	29.43% (28,297/96137)	< 0.001
Race				
Caucasian (only)	76.84% (115,013/149671)	80.52% (43,106/53534)	74.80% (71,907/96137)	< 0.001
African American (only)	14.88% (22,266/149671)	10.23% (5479/53534)	17.46% (16,787/96137)	< 0.001
AIAN (only)	1.25% (1870/149671)	1.43% (765/53534)	1.15% (1105/96137)	< 0.001
Asian (only)	4.77% (7134/149671)	5.31% (2841/53534)	4.47% (4293/96137)	< 0.001
NHOPI (only)	0.31% (466/149671)	0.31% (166/53534)	0.31% (300/96137)	0.948
More than one race	1.95% (2922/149671)	2.20% (1177/53534)	1.82% (1745/96137)	< 0.001
Neonatal characteristics				
Gestational age (weeks)	39 (38–40)	39 (38–39)	39 (38–40)	< 0.001
Birth weight (grams)	3310 (2990–3629)	3280 (2970–3593)	3317 (3005–3640)	< 0.001
Birth weight (centiles)	42.76 (19.17–71.78)	41.70 (18.40–70.53)	43.50 (19.52–72.87)	< 0.001
Birth weight (centiles)				
0th centile	14.17% (21,182/149,473)	14.32% (7654/53,456)	14.09% (13,528/96017)	0.223
10-49th centile	42.53% (63,564/149,473)	43.43% (23,215/53,456)	42.02% (40,349/96,017)	< 0.001
50–90th centile	32.12% (48,016/149,473)	31.75% (16,973/53,456)	32.33% (31,043/96,017)	0.021
> 90 centile	11.18% (16,711/149,473)	10.50% (5614/53,456)	11.56% (11,097/96,017)	< 0.001
Neonatal sex				
Female	51.34% (76,843/149,671)	53.27% (28,517/53,534)	50.27% (48,326/96,137)	< 0.001
Male	48.66% (72,828/149,671)	46.73% (25,017/53,534)	49.73% (47,811/96,137)	< 0.001
Pregnancy characteristics				
Successful ECV	64.23% (96,137/149,671)			
Fetal presentation at delivery				
Cephalic	67.96% (101,720/149,671)	24.73% (13,241/53,534)	92.03% (88,479/96,137)	< 0.001
Breech	27.05% (40,482/149,671)	70.33% (37,653/53,534)	2.94% (2829/96,137)	< 0.001
Other	2.41% (3604/149,671)	4.36% (2334/53,534)	1.32% (1270/96,137)	< 0.001
Unknown	2.58% (3865/149,671)	0.57% (306/53,534)	3.70% (3559/96,137)	< 0.001
Mode of delivery				
Spontaneous	49.47% (74,045/149671)	9.68% (5184/53534)	71.63% (68,861/96137)	< 0.001
Forceps	0.72% (1074/149671)	0.22% (120/53,534)	0.99% (954/96,137)	< 0.001
Vacuum	2.52% (3778/149,671)	0.53% (282/53,534)	3.64% (3496/96,137)	< 0.001
Cesarean	47.22% (70,679/149,671)	89.53% (47,931/53,534)	23.66% (22,748/96,137)	< 0.001
Unknown	0.06% (95/149,671)	0.03% (17/53,534)	0.08% (78/96,137)	< 0.001

*AIAN* American Indian and Alaska Native, *BMI* body mass index, *ECV* external cephalic version, *NHOPI* Native Hawaiian and Other Pacific Islanders

*Difference between failed and successful ECV

**Fig. 3:**
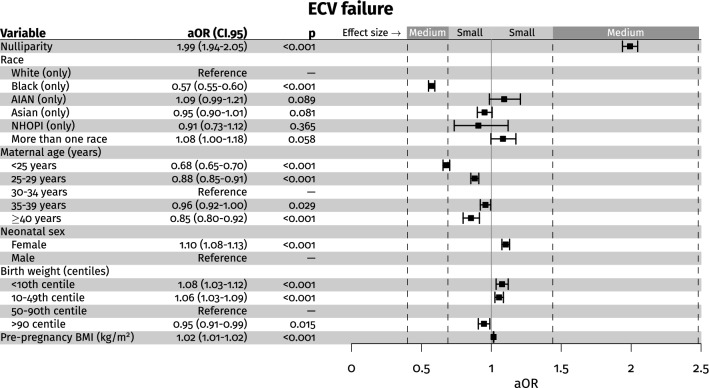
The chart exhibits the multivariate logistic regression analysis (dependent variable failed ECV). The plot shows the adjusted odds ratio (aOR), its 95% confidence interval (CI 95), and *p* value. The reported effects size magnitude is based on Cohen’s effect size interpretation [51]

Table 1 shows the differences between singleton pregnancies with a successful ECV vs. failed ECV. Table 2 and Fig. 3 show the univariate and multivariate logistic regression without interactions. Figure 3 shows the model without imputation of the missing values and considering the pre-pregnancy BMI. In these models, women younger than 29 and older than 34, the African American race, and high fetal weight centile were protective against ECV failure. Meanwhile, nulliparity, female fetal sex, and low fetal weight centile were risk factors for failed ECV. The same pattern was observed in the model with the imputed values (Supplemental Table 1). In these models (with imputed random values instead of the missings), we observed the AIAN and more than one race categories as risk factors for ECV failure. In Supplemental Table 1, we show also the multivariate models with significant interactions. According to this model, women younger than 25, older than 40, and African American women were protective factors. Additionally, fetal female sex, pre-pregnancy BMI, nulliparity, and low fetal weight centile were risk factors for failed ECV. According to this model, which considers interactions, the AIAN race was protective (OR 0.63 CI.95 0.37–1.07). In particular, considering in a non-imputed model only pre-pregnancy BMI and ethnicity, we found that in univariate analysis, AIAN was a significant risk factor for ECV failure (OR 1.15, CI 95 1.05–1.27, *p* = 0.002) but in multivariate analysis resulted in being an important protective factor (OR 0.49, CI 95 0.32–0.75, *p* = 0.001). The increased risk was significantly mediated by an interaction with pre-pregnancy BMI (interaction term pre-pregnancy BMI (km/m^2^):race AIAN OR 1.02, CI 95 1.01–1.04, *p* < 0.001). In Supplemental Table 1D, the same pattern of significant differences was observed. Supplemental Tables 1E–H show the models considering the BMI at the delivery time. The same differences were also observed in these models, and BMI at delivery was also a significant risk factor for ECV failure. Supplemental Tables 1G and H show the models with the interaction terms. In the model without imputation, where 61.99% of the BMI values were missings, female neonatal sex was a non-significant risk factor, and two significant interactions justified the increased risk of ECV failure associated with female fetal sex, found in the model without interaction terms. In particular, there was a significant interaction between young maternal age and female neonatal sex and between nulliparity and female neonatal sex (Supplemental Table 2C). However, in supplemental Table 2D with the imputation of missing values, fetal neonatal sex was a significant risk factor for ECV failure. Considering that the BMI at delivery missings were 61.99% of the assessed cohort, the models were evaluated with 1000 different random imputations, and the *p* values of the 1000 models with the interaction terms were constantly significant for the female fetal sex as a risk factor for failed ECV. Although not considered in the multivariate models, because of the high rate of missing values, weight gain during pregnancy was also associated with an increased ECV failure (OR 1.01, CI 95 1.01–1.01, *p* < 0.001) (Table 1). Considering the false discovery rate adjusted p-values, the same pattern of significant differences was observed (Supplemental Table 1).

**Table 2:** Univariate logistic regression analysis (dependent variable failed ECV)

	OR (CI 95)	*p*
Nulliparity	1.70 (1.66–1.74)	< 0.001
Race		
Caucasian (only)	Reference	1
African American (only)	0.54 (0.53–0.56)	< 0.001
AIAN (only)	1.15 (1.05–1.27)	0.002
Asian (only)	1.10 (1.05–1.16)	< 0.001
NHOPI (only)	0.92 (0.76–1.12)	0.409
More than one race	1.13 (1.04–1.21)	0.002
Maternal age (years)		
< 25 years	0.70 (0.68–0.72)	< 0.001
25–29 years	0.88 (0.86–0.91)	< 0.001
30–34 years	Reference	1
35–39 years	0.93 (0.90–0.96)	< 0.001
≥ 40 years	0.84 (0.79–0.89)	< 0.001
Neonatal sex		
Female	1.13 (1.10–1.15)	< 0.001
Male	Reference	1
Birth weight (centiles)		
< 10th centile	1.03 (1.00–1.07)	0.047
10–49th centile	1.05 (1.03–1.08)	< 0.001
50–90th centile	Reference	1
> 90 centile	0.93 (0.89–0.96)	< 0.001
Pre-pregnancy BMI (kg/m^2^)	1.01 (1.00–1.01)	< 0.001
BMI at delivery (kg/m^2^)	1.01 (1.01–1.01)	< 0.001

*AIAN* American Indian and Alaska Native, *BMI* body mass index, *ECV* external cephalic version, *NHOPI* Native Hawaiian and Other Pacific Islanders

## Discussion

### Main findings

We found that 64.23% of the procedures were successful. Moreover, among the successful ECV procedures, the majority were delivered vaginally and only 23.66% delivered by CS. The prevalence of spontaneous vaginal delivery in successful ECV was 71.63%. Among the failed ECV procedures, 24.74% had a cephalic presentation at delivery, but 63.11% of these pregnancies were delivered by CS. Nulliparity, female sex, low fetal weight centile (10–49th centile and small for gestational age fetuses < 10th centile), high pre-pregnancy BMI, high BMI at delivery, and high weight gain during pregnancy were associated with an increased ECV failure. African American, AIAN race categories, younger maternal age (< 25 years), and older maternal age (> 40 years) were protective factors against ECV failure.

### Results in the context of what is known

Implementing ECV procedures seems feasible and, in case of a successful procedure, brings high chances of delivering spontaneously and vaginally. Hence reducing the burden related to the high rate of CS in breech presenting fetuses at delivery [2–5]. In the previous literature, the prevalence of CS after a successful ECV was 18.71% (CI 95 13.01–26.16%), which is similar to the prevalence found in our study 23.66% [25]. Moreover, women with a failed procedure can be reassured that in 8.48% of the cases, fetuses are presenting anyway cephalic, and the delivery can be a vaginal spontaneous cephalic delivery (Fig. 2A and B). Recently Birene and coworkers found that performing ECV did not reduce the number of CS [26]. However, in their control group, 33% of pregnancies had breech vaginal delivery [26]. Our study found that vaginal breech delivery is performed only in 1.06% after the failed ECV procedures and in 0.42% after the successful procedures.

The reasons that can lead to ECV failure can be multiple. In addition to the mechanical difficulty during the ECV maneuvers, other reasons related to ECV failure are the spontaneous repositioning of the fetus in the breech position and the occurrence of complications (e.g., placental abruption, vaginal bleeding, or abnormal cardiotocography) [27]. Although the overall prevalence of serious complications is low, abnormal fetal heartbeat, including bradycardia, are among the most frequent reasons to discontinue the procedure [27].

#### Nulliparity

In the previous literature, parity was consistently associated with ECV failure or success, and in particular, nulliparity was associated with ECV failure [13–16, 25, 28–32]. Our data confirm the strong association between nulliparity and ECV procedure failure. Probably the difference in comparison to other risk factors, which showed varying outcomes, is the magnitude of the effect size of parity that allows a better classification of the association also with small sample sizes.

#### Female fetal sex

Even though many different explanations related to the sex differences between females and males are possible, there is no definitive answer. It is known that there are significant anthropometric differences between female and male fetuses. Female fetuses have significantly shorter crown-rump, crown-heel distances, and lower weight than males [33]. They also have a higher relative fat content in respect to the muscle mass [33]. This different ratio between muscle and fat masses can lead to different behaviors during the ECV maneuvers, and it can explain the differences in the ECV success rate. Even, López-Pérez and coworkers found an increased rate of ECV failures associated with female neonatal sex [16].

#### Fetal weight

Previous studies found a higher prevalence of fetal weight < 2500 g and lower birth weight or estimated fetal weight in the groups of failed ECV procedures [14, 30]. We found a significant correlation between low fetal weight centile (10-49th centile and small for gestational age fetuses < 10th centile) and ECV procedure failure confirming that a low fetal weight can impair the procedure’s success. We also found an increased ECV success rate in fetal weight > 90th centile compared to the reference category. However, this difference was mainly non-significant after adjusting for the false discovery rate test in the models with the interactions. The only significant difference was found in the model considering the BMI at delivery and comprising the imputed values. However, further evidence is required because of this model’s high number of imputed BMI values.

#### Maternal anthropometric characteristics

This study confirms high maternal BMI is a factor that impedes the ECV success [13, 15, 17, 18, 34]. In previous literature, there was a broad heterogeneity in the way to consider maternal anthropometric features in the prediction of ECV success [15]. Also, the results were heterogeneous [6]. Some authors found that maternal weight was not a significant predictor of ECV failure or success [16, 30, 31, 35–37]. Meanwhile, two recent studies found that an increased pre-pregnancy BMI was a significant risk factor for ECV failure [17, 34]. Moreover, the high maternal BMI at the time of the procedure was also found to be a significant predictor of ECV failure [34]. To our knowledge, only limited data are presented in previous literature about the association between weight gain during pregnancy and ECV failure. López-Pérez and coworkers showed a non-significant higher weight gain among the group of pregnancies with a successful ECV procedure. Otherwise, we found an increased risk of failure associated with high weight gain during pregnancy. However, this study can not be conclusive because of this factor’s high number of missings. Furthermore, the BMI at delivery or at the time of the procedure partly also considers the effect of weight gain during pregnancy, and our results are in accordance with the findings of Dong and coworkers [34].

#### Maternal race

We also found that the African American race to influence the ECV success positively. Previous studies hypothesized that the increased chance of ECV success in the African American race was because presenting part usually remains high until the onset of labor [18, 19]. Hence, the high presenting part facilitates the ECV maneuvers. Also, AIAN was found to be a favoring factor for successful ECV. The AIAN group is also known to have a lower incidence of CS than the other race strata [38].

#### Maternal age

The previous literature found no significant correlation between maternal age and ECV success or failure. It was only found that older maternal age is a significant risk factor for breech presentation [39]. Furthermore, Dong and coworkers found that mothers younger than 35 significantly correlated to a vaginal delivery after a successful ECV procedure [34]. Our study found a U-shape association between maternal age and ECV success. In particular, younger maternal age (< 25 years) and older maternal age (> 40 years) were associated with an increased rate of ECV success. The increased success rate in younger women can be related to the reduction of soft tissue elasticity correlated with older women’s age [40]. It is also known that the increased rigidity of the abdominal and uterine wall was found to be a risk factor for ECV failure [15, 41]. However, this cannot explain the reduced risk in women older than 40. In this case, we believe that a possible explanation can be a more stringent selection of candidates for the procedure in this age group. In addition, there are many significant interaction factors with maternal age. For example, a mother younger than 25 years carrying a female fetus will have an odds of 0.81 instead of 0.65 (Supplemental Table 2). Furthermore, a mother younger than 25 years and nulliparous will have an odds of 1.10 instead of 0.65 (Supplemental Table 2).

### Strengths and weaknesses

The main strength of this study is the vast observational cohort analyzed. However, several potential limitations should be discussed. The retrospective nature of this study limits the value of the results because of the possible inherited biases from the data collection planning and procedures.

The absence of information about methodological issues of the procedures limits the study. It was impossible to correct for some factors known to be correlated to the procedure’s success. Gestational age at the procedure was not available, and it was previously found to be a significant predictive factor for the procedure success [34]. However, in the US, it is suggested for the recruitment and procedure timing the gestational period between 36 and 37 weeks and 6 days [6]. This period is within a relatively small timeframe and is associated with a high probability of procedure success [34]. Another critical issue is the unavailable information about the operator’s experience that is known to favor the procedure’s success [42]. Repeated procedures were not recorded. The practice of repeating procedures can further reduce the number of breech fetuses at delivery [43]. However, due to the broad cohort included and the nationwide nature of the study, it is unlikely that these features will influence the actual results of our analysis. Another missing information in the original dataset was the time interval between the ECV procedure and the delivery. However, it is unlikely that this factor will alter the results of our analysis as in previous literature, was found no significant association between the time-interval and the mode of delivery [44].

In addition to information on the methodology of the technique, other information was missing. The estimated fetal weight at the time of the procedure was not recorded. As this is an essential factor in predicting the procedure failure or success, we assumed that the same weight centile registered at birth was effectively the weight centile at the procedure time. In addition, according to this assumption, we used a prenatal growth curve to assess fetal centiles. In all our analyses, including the multivariate models, we considered the fetal/newborn weight centile. Also, the estimated amount of amniotic fluid was not recorded in the original dataset. Previous studies indicate an association between amniotic fluid quantity and successful ECV [13, 14, 16, 18, 34–36, 45, 46], whereas additional studies do not [30, 37, 47]. Due to the heterogeneity of the literature about this issue and the possible errors related to the ultrasound estimation of the amniotic fluid quantity, it is unlikely that this information would have changed the results of our analysis. Even the placenta location was not present in the analyzed dataset. Some previous reports showed an association between successful ECV and placenta location [13, 19, 30, 32, 34], but others failed to see an association [16, 35–37, 47]. Other factors were not present in the original dataset, such as the characteristics of the maternal abdominal and uterine wall [15, 41, 48], the persistence of breech presentation [49], the type of presentation at the time of ECV maneuver [13, 18, 47], and the engagement of the fetal presenting part into the pelvis [15, 16, 18, 50]. Moreover, the association between ECV failure and female fetal sex reflects a small effect size. Generally, a Cohen h effect size is defined as small as 0.20; in this case, the observed effect size of female fetal sex is 0.06 [51]. Previous literature developed different predictive instruments for ECV failure or success with an accuracy ranging from 70 to 80% [16, 34]. However, these models were developed using small cohorts underpowered to identify potential predictors with a small effect size that can improve the model’s discrimination capacity. The advantage of this cohort is to better depict the effect size of some parameters such as BMI, maternal weight gain during pregnancy, or fetal sex, which can be useful to plan future studies to develop accurate prediction algorithms for ECV success or failure.

### Generalisability, relevance of the findings, and unanswered questions

Accurate knowledge of the factors involved in the ECV procedure and subsequent delivery is fundamental in making appropriate counseling for women who access this service. Moreover, these results increase the knowledge about the factors involved in ECV success or failure. Greater knowledge means being able to face this path in the best and most conscious way, which can reduce the number of cesarean sections, thus reducing the subsequent morbidity related to them.

## Conclusions

This study found a high prevalence of successful ECV procedures and spontaneous vaginal delivery after successful ECV procedures. Furthermore, the present results found nulliparity, female fetal sex, low fetal weight centile, high pre-pregnancy BMI, high BMI at delivery, and increased maternal weight gain during pregnancy negatively correlated to ECV success. African American women and AIAN race categories were significant protective factors against ECV failure. Maternal age presented a U-shape risk profile, with younger and older maternal ages found to be significant protective factors against ECV failure. These findings can potentially improve the knowledge about the factors involved in ECV, allowing more informed counseling to the women undergoing this procedure.

## Supplementary Information

Below is the link to the electronic supplementary material.

Supplementary file1 (DOCX 32 KB)

## Data Availability

All datasets are freely available at https://www.cdc.gov/nchs/data_access/Vitalstatsonline.htm.

## Abbreviations

AIAN: American Indian and Alaska Native
BMI: Body mass index
CI.95: 95% Confidence interval
CS: Cesarean section
ECV: External cephalic version
IQR: Interquartile range
NHOPI: Native Hawaiian and Other Pacific Islanders
OR: Odds ratio
STROBE: Strengthening the Reporting of Observational Studies in Epidemiology
US: United States
