# Supplementary material for: Factors influencing the effect of external cephalic version: a retrospective nationwide cohort analysis
Source: Arch Gynecol Obstet. 2022 Sep 7;308(4):1127–37. doi: 10.1007/s00404-022-06763-2 (PMC10435405; doi:10.1007/s00404-022-06763-2)
Supplement: Supplementary file 1 — Supplementary file1 (DOCX 32 KB) [file 404_2022_6763_MOESM1_ESM.docx]

**Supplemental Table 1**- Univariate and multivariate (*) logistic regression analysis (dependent variable failed ECV). The multivariate logistic regression models B, C, E, and F also show significant interaction terms. False discovery rate adjustment was applied to the p-values (**). Part A shows the model that included pre-pregnancy BMI without interaction terms and imputing missing values. Part B shows the model that included pre-pregnancy BMI without interaction terms but with random imputation of missing values. Part C shows the model that had pre-pregnancy BMI without imputation of the missing values. Part D shows the model that included pre-pregnancy BMI with random imputation of the missing values. Part E shows the model with BMI at delivery without interaction terms and missing values imputation. Part F shows the model with BMI at delivery without interaction terms but with random imputation of missing values. Part G shows the model that included BMI at delivery without imputation of the missing values. Part H shows the model that included BMI at delivery with random imputation of the missing values.

|  | **OR (CI.95)** | **p** | **OR (CI.95)(*)** | **p(*)** | **p(**)** |
| --- | --- | --- | --- | --- | --- |
| **A) Pre-pregnancy BMI, no imputation, no interaction** |  |  |  |  |  |
| Nulliparity | 1.70 (1.66 - 1.74) | <0.001 | 1.99 (1.94 - 2.05) | <0.001 | <0.001 |
| Race |  |  |  |  |  |
| White (only) | Reference | 1.000 | Reference | 1.000 |  |
| Black (only) | 0.54 (0.53 - 0.56) | <0.001 | 0.57 (0.55 - 0.60) | <0.001 | <0.001 |
| AIAN (only) | 1.15 (1.05 - 1.27) | 0.002 | 1.09 (0.99 - 1.21) | 0.089 | 0.095 |
| Asian (only) | 1.10 (1.05 - 1.16) | <0.001 | 0.95 (0.90 - 1.01) | 0.081 | 0.094 |
| NHOPI (only) | 0.92 (0.76 - 1.12) | 0.409 | 0.91 (0.73 - 1.12) | 0.365 | 0.365 |
| More than one race | 1.13 (1.04 - 1.21) | 0.002 | 1.08 (1.00 - 1.18) | 0.058 | 0.072 |
| Maternal age (years) |  |  |  |  |  |
| <25 years | 0.70 (0.68 - 0.72) | <0.001 | 0.68 (0.65 - 0.70) | <0.001 | <0.001 |
| 25-29 years | 0.88 (0.86 - 0.91) | <0.001 | 0.88 (0.85 - 0.91) | <0.001 | <0.001 |
| 30-34 years | Reference | 1.000 | Reference | 1.000 |  |
| 35-39 years | 0.93 (0.90 - 0.96) | <0.001 | 0.96 (0.92 - 1.00) | 0.029 | 0.040 |
| ≥40 years | 0.84 (0.79 - 0.89) | <0.001 | 0.85 (0.80 - 0.92) | <0.001 | <0.001 |
| Neonatal sex |  |  |  |  |  |
| Female | 1.13 (1.10 - 1.15) | <0.001 | 1.10 (1.08 - 1.13) | <0.001 | <0.001 |
| Male | Reference | 1.000 | Reference | 1.000 |  |
| Birth weight (centiles) |  |  |  |  |  |
| <10th centile | 1.03 (1.00 - 1.07) | 0.047 | 1.08 (1.03 - 1.12) | <0.001 | <0.001 |
| 10-49th centile | 1.05 (1.03 - 1.08) | <0.001 | 1.06 (1.03 - 1.09) | <0.001 | <0.001 |
| 50-90th centile | Reference | 1.000 | Reference | 1.000 |  |
| >90 centile | 0.93 (0.89 - 0.96) | <0.001 | 0.95 (0.91 - 0.99) | 0.015 | 0.022 |
| Pre-pregnancy BMI (kg/m²) | 1.01 (1.00 - 1.01) | <0.001 | 1.02 (1.01 - 1.02) | <0.001 | <0.001 |
| **B) Pre-pregnancy BMI, imputation, no interaction** |  |  |  |  |  |
| Nulliparity | 1.70 (1.66 - 1.74) | <0.001 | 1.90 (1.86 - 1.95) | <0.001 | <0.001 |
| Race |  |  |  |  |  |
| White (only) | Reference | 1.000 | Reference | 1.000 |  |
| Black (only) | 0.54 (0.53 - 0.56) | <0.001 | 0.58 (0.56 - 0.60) | <0.001 | <0.001 |
| AIAN (only) | 1.15 (1.05 - 1.27) | 0.002 | 1.27 (1.16 - 1.39) | <0.001 | <0.001 |
| Asian (only) | 1.10 (1.05 - 1.16) | <0.001 | 1.01 (0.96 - 1.06) | 0.670 | 0.718 |
| NHOPI (only) | 0.92 (0.76 - 1.12) | 0.409 | 0.99 (0.82 - 1.20) | 0.953 | 0.953 |
| More than one race | 1.13 (1.04 - 1.21) | 0.002 | 1.16 (1.08 - 1.26) | <0.001 | <0.001 |
| Maternal age (years) |  |  |  |  |  |
| <25 years | 0.70 (0.68 - 0.72) | <0.001 | 0.62 (0.60 - 0.64) | <0.001 | <0.001 |
| 25-29 years | 0.88 (0.86 - 0.91) | <0.001 | 0.85 (0.83 - 0.88) | <0.001 | <0.001 |
| 30-34 years | Reference | 1.000 | Reference | 1.000 |  |
| 35-39 years | 0.93 (0.90 - 0.96) | <0.001 | 0.99 (0.95 - 1.02) | 0.423 | 0.488 |
| ≥40 years | 0.84 (0.79 - 0.89) | <0.001 | 0.92 (0.87 - 0.98) | 0.007 | 0.011 |
| Neonatal sex |  |  |  |  |  |
| Female | 1.13 (1.10 - 1.15) | <0.001 | 1.11 (1.09 - 1.14) | <0.001 | <0.001 |
| Male | Reference | 1.000 | Reference | 1.000 |  |
| Birth weight (centiles) |  |  |  |  |  |
| <10th centile | 1.03 (1.00 - 1.07) | 0.051 | 1.05 (1.01 - 1.08) | 0.009 | 0.012 |
| 10-49th centile | 1.05 (1.03 - 1.08) | <0.001 | 1.04 (1.01 - 1.07) | 0.002 | 0.003 |
| 50-90th centile | Reference | 1.000 | Reference | 1.000 |  |
| >90 centile | 0.92 (0.89 - 0.96) | <0.001 | 0.96 (0.92 - 1.00) | 0.032 | 0.040 |
| Pre-pregnancy BMI (kg/m²) | 1.01 (1.00 - 1.01) | <0.001 | 1.01 (1.01 - 1.01) | <0.001 | <0.001 |
| **C) Pre-pregnancy BMI, no imputation, interaction** |  |  |  |  |  |
| Nulliparity | 1.70 (1.66 - 1.74) | <0.001 | 2.67 (2.49 - 2.87) | <0.001 | <0.001 |
| Race |  |  |  |  |  |
| White (only) | Reference | 1.000 | Reference | 1.000 |  |
| Black (only) | 0.54 (0.53 - 0.56) | <0.001 | 0.42 (0.35 - 0.51) | <0.001 | <0.001 |
| AIAN (only) | 1.15 (1.05 - 1.27) | 0.002 | 0.63 (0.37 - 1.07) | 0.086 | 0.250 |
| Asian (only) | 1.10 (1.05 - 1.16) | <0.001 | 0.84 (0.61 - 1.14) | 0.253 | 0.549 |
| NHOPI (only) | 0.92 (0.76 - 1.12) | 0.409 | 0.57 (0.20 - 1.68) | 0.312 | 0.561 |
| More than one race | 1.13 (1.04 - 1.21) | 0.002 | 1.10 (0.75 - 1.63) | 0.625 | 0.811 |
| Maternal age (years) |  |  |  |  |  |
| <25 years | 0.70 (0.68 - 0.72) | <0.001 | 0.65 (0.55 - 0.76) | <0.001 | <0.001 |
| 25-29 years | 0.88 (0.86 - 0.91) | <0.001 | 0.92 (0.79 - 1.08) | 0.298 | 0.555 |
| 30-34 years | Reference | 1.000 | Reference | 1.000 |  |
| 35-39 years | 0.93 (0.90 - 0.96) | <0.001 | 0.97 (0.81 - 1.17) | 0.755 | 0.823 |
| ≥40 years | 0.84 (0.79 - 0.89) | <0.001 | 0.63 (0.45 - 0.87) | 0.005 | 0.025 |
| Neonatal sex |  |  |  |  |  |
| Female | 1.13 (1.10 - 1.15) | <0.001 | 1.10 (1.04 - 1.17) | 0.002 | 0.009 |
| Male | Reference | 1.000 | Reference | 1.000 |  |
| Birth weight (centiles) |  |  |  |  |  |
| <10th centile | 1.03 (1.00 - 1.07) | 0.047 | 1.74 (1.44 - 2.09) | <0.001 | <0.001 |
| 10-49th centile | 1.05 (1.03 - 1.08) | <0.001 | 1.41 (1.23 - 1.61) | <0.001 | <0.001 |
| 50-90th centile | Reference | 1.000 | Reference | 1.000 |  |
| >90 centile | 0.93 (0.89 - 0.96) | <0.001 | 0.90 (0.73 - 1.09) | 0.280 | 0.553 |
| Pre-pregnancy BMI (kg/m²) | 1.01 (1.00 - 1.01) | <0.001 | 1.02 (1.01 - 1.02) | <0.001 | <0.001 |
| Nulliparity:Race Black (only) |  |  | 0.75 (0.68 - 0.82) | <0.001 | <0.001 |
| Nulliparity:Race AIAN (only) |  |  | 1.17 (0.90 - 1.52) | 0.239 | 0.549 |
| Nulliparity:Race Asian (only) |  |  | 0.88 (0.78 - 0.99) | 0.030 | 0.105 |
| Nulliparity:Race NHOPI (only) |  |  | 1.37 (0.78 - 2.40) | 0.270 | 0.553 |
| Nulliparity:Race More than one Race |  |  | 0.89 (0.74 - 1.07) | 0.203 | 0.503 |
| Nulliparity:Maternal age (years) <25 years |  |  | 0.64 (0.60 - 0.69) | <0.001 | <0.001 |
| Nulliparity:Maternal age (years) 25-29 years |  |  | 0.89 (0.83 - 0.96) | 0.002 | 0.009 |
| Nulliparity:Maternal age (years) 35-39 years |  |  | 0.93 (0.85 - 1.03) | 0.166 | 0.422 |
| Nulliparity:Maternal age (years) ≥40 years |  |  | 0.86 (0.71 - 1.05) | 0.141 | 0.369 |
| Nulliparity:Neonatal sex Female |  |  | 1.05 (1.00 - 1.11) | 0.058 | 0.188 |
| Nulliparity:Birth weight (centiles) <10th centile |  |  | 0.67 (0.62 - 0.73) | <0.001 | <0.001 |
| Nulliparity:Birth weight (centiles) 10-49th centile |  |  | 0.89 (0.84 - 0.95) | <0.001 | 0.003 |
| Nulliparity:Birth weight (centiles) >90 centile |  |  | 0.96 (0.86 - 1.06) | 0.431 | 0.646 |
| Race Black (only):Maternal age (years) <25 years |  |  | 1.10 (0.99 - 1.23) | 0.078 | 0.233 |
| Race AIAN (only):Maternal age (years) <25 years |  |  | 0.89 (0.66 - 1.21) | 0.456 | 0.660 |
| Race Asian (only):Maternal age (years) <25 years |  |  | 1.14 (0.93 - 1.40) | 0.211 | 0.507 |
| Race NHOPI (only):Maternal age (years) <25 years |  |  | 1.42 (0.75 - 2.72) | 0.283 | 0.553 |
| Race More than one Race:Maternal age (years) <25 years |  |  | 0.99 (0.79 - 1.25) | 0.952 | 0.984 |
| Race Black (only):Maternal age (years) 25-29 years |  |  | 1.04 (0.93 - 1.17) | 0.450 | 0.660 |
| Race AIAN (only):Maternal age (years) 25-29 years |  |  | 1.11 (0.83 - 1.50) | 0.476 | 0.678 |
| Race Asian (only):Maternal age (years) 25-29 years |  |  | 1.07 (0.92 - 1.24) | 0.362 | 0.605 |
| Race NHOPI (only):Maternal age (years) 25-29 years |  |  | 1.15 (0.63 - 2.12) | 0.645 | 0.811 |
| Race More than one Race:Maternal age (years) 25-29 years |  |  | 0.89 (0.71 - 1.12) | 0.320 | 0.561 |
| Race Black (only):Maternal age (years) 35-39 years |  |  | 1.03 (0.89 - 1.18) | 0.724 | 0.811 |
| Race AIAN (only):Maternal age (years) 35-39 years |  |  | 0.98 (0.69 - 1.40) | 0.917 | 0.963 |
| Race Asian (only):Maternal age (years) 35-39 years |  |  | 1.03 (0.89 - 1.20) | 0.664 | 0.811 |
| Race NHOPI (only):Maternal age (years) 35-39 years |  |  | 0.86 (0.41 - 1.80) | 0.680 | 0.811 |
| Race More than one Race:Maternal age (years) 35-39 years |  |  | 0.80 (0.61 - 1.06) | 0.117 | 0.328 |
| Race Black (only):Maternal age (years) ≥40 years |  |  | 1.30 (1.04 - 1.62) | 0.021 | 0.076 |
| Race AIAN (only):Maternal age (years) ≥40 years |  |  | 0.74 (0.36 - 1.54) | 0.424 | 0.646 |
| Race Asian (only):Maternal age (years) ≥40 years |  |  | 1.12 (0.87 - 1.43) | 0.376 | 0.607 |
| Race NHOPI (only):Maternal age (years) ≥40 years |  |  | 0.69 (0.16 - 2.89) | 0.607 | 0.811 |
| Race More than one Race:Maternal age (years) ≥40 years |  |  | 1.08 (0.68 - 1.72) | 0.734 | 0.811 |
| Race Black (only):Neonatal sex Female |  |  | 0.89 (0.83 - 0.96) | 0.004 | 0.020 |
| Race AIAN (only):Neonatal sex Female |  |  | 0.89 (0.72 - 1.09) | 0.252 | 0.549 |
| Race Asian (only):Neonatal sex Female |  |  | 0.98 (0.87 - 1.09) | 0.693 | 0.811 |
| Race NHOPI (only):Neonatal sex Female |  |  | 0.81 (0.53 - 1.25) | 0.342 | 0.586 |
| Race More than one Race:Neonatal sex Female |  |  | 1.00 (0.84 - 1.17) | 0.961 | 0.984 |
| Race Black (only):Pre-pregnancy BMI (kg/m²) |  |  | 1.01 (1.01 - 1.02) | <0.001 | <0.001 |
| Race AIAN (only):Pre-pregnancy BMI (kg/m²) |  |  | 1.02 (1.01 - 1.04) | 0.007 | 0.032 |
| Race Asian (only):Pre-pregnancy BMI (kg/m²) |  |  | 1.01 (0.99 - 1.02) | 0.368 | 0.605 |
| Race NHOPI (only):Pre-pregnancy BMI (kg/m²) |  |  | 1.01 (0.98 - 1.04) | 0.406 | 0.644 |
| Race More than one Race:Pre-pregnancy BMI (kg/m²) |  |  | 1.00 (0.99 - 1.02) | 0.620 | 0.811 |
| Maternal age (years) <25 years:Neonatal sex Female |  |  | 1.13 (1.05 - 1.21) | 0.001 | 0.007 |
| Maternal age (years) 25-29 years:Neonatal sex Female |  |  | 1.01 (0.95 - 1.08) | 0.708 | 0.811 |
| Maternal age (years) 35-39 years:Neonatal sex Female |  |  | 0.98 (0.90 - 1.06) | 0.577 | 0.808 |
| Maternal age (years) ≥40 years:Neonatal sex Female |  |  | 0.93 (0.81 - 1.07) | 0.320 | 0.561 |
| Maternal age (years) <25 years:Birth weight (centiles) <10th centile |  |  | 0.98 (0.88 - 1.09) | 0.722 | 0.811 |
| Maternal age (years) 25-29 years:Birth weight (centiles) <10th centile |  |  | 0.88 (0.79 - 0.98) | 0.018 | 0.070 |
| Maternal age (years) 35-39 years:Birth weight (centiles) <10th centile |  |  | 1.03 (0.91 - 1.18) | 0.627 | 0.811 |
| Maternal age (years) ≥40 years:Birth weight (centiles) <10th centile |  |  | 1.01 (0.81 - 1.27) | 0.903 | 0.960 |
| Maternal age (years) <25 years:Birth weight (centiles) 10-49th centile |  |  | 0.95 (0.88 - 1.04) | 0.255 | 0.549 |
| Maternal age (years) 25-29 years:Birth weight (centiles) 10-49th centile |  |  | 0.97 (0.90 - 1.05) | 0.425 | 0.646 |
| Maternal age (years) 35-39 years:Birth weight (centiles) 10-49th centile |  |  | 1.05 (0.96 - 1.15) | 0.269 | 0.553 |
| Maternal age (years) ≥40 years:Birth weight (centiles) 10-49th centile |  |  | 0.97 (0.83 - 1.14) | 0.726 | 0.811 |
| Maternal age (years) <25 years:Birth weight (centiles) >90 centile |  |  | 1.13 (0.99 - 1.28) | 0.067 | 0.209 |
| Maternal age (years) 25-29 years:Birth weight (centiles) >90 centile |  |  | 1.00 (0.89 - 1.12) | 0.977 | 0.989 |
| Maternal age (years) 35-39 years:Birth weight (centiles) >90 centile |  |  | 0.97 (0.85 - 1.11) | 0.661 | 0.811 |
| Maternal age (years) ≥40 years:Birth weight (centiles) >90 centile |  |  | 0.96 (0.77 - 1.19) | 0.694 | 0.811 |
| Maternal age (years) <25 years:Pre-pregnancy BMI (kg/m²) |  |  | 1.01 (1.00 - 1.01) | 0.008 | 0.034 |
| Maternal age (years) 25-29 years:Pre-pregnancy BMI (kg/m²) |  |  | 1.00 (0.99 - 1.01) | 0.989 | 0.989 |
| Maternal age (years) 35-39 years:Pre-pregnancy BMI (kg/m²) |  |  | 1.00 (0.99 - 1.01) | 0.886 | 0.955 |
| Maternal age (years) ≥40 years:Pre-pregnancy BMI (kg/m²) |  |  | 1.01 (1.00 - 1.02) | 0.012 | 0.050 |
| Neonatal sex Female:Birth weight (centiles) <10th centile |  |  | 0.94 (0.86 - 1.02) | 0.124 | 0.336 |
| Neonatal sex Female:Birth weight (centiles) 10-49th centile |  |  | 0.99 (0.93 - 1.04) | 0.611 | 0.811 |
| Neonatal sex Female:Birth weight (centiles) >90 centile |  |  | 0.91 (0.83 - 1.00) | 0.042 | 0.140 |
| Birth weight (centiles) <10th centile:Pre-pregnancy BMI (kg/m²) |  |  | 0.99 (0.98 - 1.00) | 0.002 | 0.009 |
| Birth weight (centiles) 10-49th centile:Pre-pregnancy BMI (kg/m²) |  |  | 0.99 (0.99 - 1.00) | <0.001 | <0.001 |
| Birth weight (centiles) >90 centile:Pre-pregnancy BMI (kg/m²) |  |  | 1.00 (1.00 - 1.01) | 0.297 | 0.555 |
| **D) Pre-pregnancy BMI, imputation, interaction** |  |  |  |  |  |
| Nulliparity | 1.70 (1.66 - 1.74) | <0.001 | 3.23 (2.88 - 3.62) | <0.001 | <0.001 |
| Race |  |  |  |  |  |
| White (only) | Reference | 1.000 | Reference | 1.000 |  |
| Black (only) | 0.54 (0.53 - 0.56) | <0.001 | 0.44 (0.37 - 0.51) | <0.001 | <0.001 |
| AIAN (only) | 1.15 (1.05 - 1.27) | 0.002 | 0.80 (0.48 - 1.33) | 0.387 | 0.641 |
| Asian (only) | 1.10 (1.05 - 1.16) | <0.001 | 1.11 (0.85 - 1.45) | 0.455 | 0.693 |
| NHOPI (only) | 0.92 (0.76 - 1.12) | 0.409 | 0.65 (0.24 - 1.72) | 0.385 | 0.641 |
| More than one race | 1.13 (1.04 - 1.21) | 0.002 | 1.08 (0.74 - 1.59) | 0.681 | 0.769 |
| Maternal age (years) |  |  |  |  |  |
| <25 years | 0.70 (0.68 - 0.72) | <0.001 | 0.63 (0.55 - 0.73) | <0.001 | <0.001 |
| 25-29 years | 0.88 (0.86 - 0.91) | <0.001 | 0.87 (0.76 - 0.99) | 0.037 | 0.138 |
| 30-34 years | Reference | 1.000 | Reference | 1.000 |  |
| 35-39 years | 0.93 (0.90 - 0.96) | <0.001 | 0.99 (0.85 - 1.17) | 0.936 | 0.956 |
| ≥40 years | 0.84 (0.79 - 0.89) | <0.001 | 0.68 (0.52 - 0.91) | 0.009 | 0.038 |
| Neonatal sex |  |  |  |  |  |
| Female | 1.13 (1.10 - 1.15) | <0.001 | 1.10 (1.05 - 1.15) | <0.001 | <0.001 |
| Male | Reference | 1.000 | Reference | 1.000 |  |
| Birth weight (centiles) |  |  |  |  |  |
| <10th centile | 1.03 (1.00 - 1.07) | 0.051 | 1.68 (1.43 - 1.97) | <0.001 | <0.001 |
| 10-49th centile | 1.05 (1.03 - 1.08) | <0.001 | 1.43 (1.27 - 1.61) | <0.001 | <0.001 |
| 50-90th centile | Reference | 1.000 | Reference | 1.000 |  |
| >90 centile | 0.92 (0.89 - 0.96) | <0.001 | 0.83 (0.70 - 0.98) | 0.028 | 0.108 |
| Pre-pregnancy BMI (kg/m²) | 1.01 (1.00 - 1.01) | <0.001 | 1.01 (1.01 - 1.02) | <0.001 | <0.001 |
| Nulliparity:Race Black (only) |  |  | 0.81 (0.75 - 0.88) | <0.001 | <0.001 |
| Nulliparity:Race AIAN (only) |  |  | 1.20 (0.94 - 1.52) | 0.146 | 0.400 |
| Nulliparity:Race Asian (only) |  |  | 0.86 (0.77 - 0.95) | 0.005 | 0.023 |
| Nulliparity:Race NHOPI (only) |  |  | 1.54 (0.93 - 2.53) | 0.092 | 0.276 |
| Nulliparity:Race More than one Race |  |  | 0.92 (0.78 - 1.09) | 0.323 | 0.589 |
| Nulliparity:Maternal age (years) <25 years |  |  | 0.66 (0.62 - 0.71) | <0.001 | <0.001 |
| Nulliparity:Maternal age (years) 25-29 years |  |  | 0.87 (0.82 - 0.93) | <0.001 | <0.001 |
| Nulliparity:Maternal age (years) 35-39 years |  |  | 0.92 (0.85 - 1.00) | 0.060 | 0.199 |
| Nulliparity:Maternal age (years) ≥40 years |  |  | 0.86 (0.73 - 1.02) | 0.084 | 0.260 |
| Nulliparity:Birth weight (centiles) <10th centile |  |  | 0.71 (0.66 - 0.76) | <0.001 | <0.001 |
| Nulliparity:Birth weight (centiles) 10-49th centile |  |  | 0.89 (0.85 - 0.95) | <0.001 | <0.001 |
| Nulliparity:Birth weight (centiles) >90 centile |  |  | 0.97 (0.89 - 1.06) | 0.561 | 0.757 |
| Nulliparity:Pre-pregnancy BMI (kg/m²) |  |  | 0.99 (0.99 - 1.00) | <0.001 | <0.001 |
| Race Black (only):Maternal age (years) <25 years |  |  | 1.03 (0.94 - 1.14) | 0.490 | 0.723 |
| Race AIAN (only):Maternal age (years) <25 years |  |  | 0.97 (0.73 - 1.27) | 0.812 | 0.866 |
| Race Asian (only):Maternal age (years) <25 years |  |  | 1.23 (1.03 - 1.48) | 0.024 | 0.096 |
| Race NHOPI (only):Maternal age (years) <25 years |  |  | 1.22 (0.67 - 2.20) | 0.520 | 0.734 |
| Race More than one Race:Maternal age (years) <25 years |  |  | 0.97 (0.79 - 1.20) | 0.797 | 0.861 |
| Race Black (only):Maternal age (years) 25-29 years |  |  | 1.02 (0.93 - 1.13) | 0.649 | 0.757 |
| Race AIAN (only):Maternal age (years) 25-29 years |  |  | 1.11 (0.84 - 1.46) | 0.470 | 0.704 |
| Race Asian (only):Maternal age (years) 25-29 years |  |  | 1.11 (0.97 - 1.26) | 0.123 | 0.347 |
| Race NHOPI (only):Maternal age (years) 25-29 years |  |  | 1.15 (0.66 - 2.00) | 0.623 | 0.757 |
| Race More than one Race:Maternal age (years) 25-29 years |  |  | 0.94 (0.76 - 1.17) | 0.591 | 0.757 |
| Race Black (only):Maternal age (years) 35-39 years |  |  | 1.03 (0.91 - 1.16) | 0.610 | 0.757 |
| Race AIAN (only):Maternal age (years) 35-39 years |  |  | 0.89 (0.64 - 1.25) | 0.513 | 0.734 |
| Race Asian (only):Maternal age (years) 35-39 years |  |  | 1.03 (0.90 - 1.18) | 0.653 | 0.757 |
| Race NHOPI (only):Maternal age (years) 35-39 years |  |  | 0.95 (0.48 - 1.88) | 0.882 | 0.910 |
| Race More than one Race:Maternal age (years) 35-39 years |  |  | 0.83 (0.64 - 1.08) | 0.157 | 0.404 |
| Race Black (only):Maternal age (years) ≥40 years |  |  | 1.15 (0.94 - 1.40) | 0.162 | 0.404 |
| Race AIAN (only):Maternal age (years) ≥40 years |  |  | 0.71 (0.36 - 1.40) | 0.325 | 0.589 |
| Race Asian (only):Maternal age (years) ≥40 years |  |  | 1.17 (0.94 - 1.47) | 0.164 | 0.404 |
| Race NHOPI (only):Maternal age (years) ≥40 years |  |  | 0.69 (0.18 - 2.61) | 0.588 | 0.757 |
| Race More than one Race:Maternal age (years) ≥40 years |  |  | 0.99 (0.64 - 1.53) | 0.977 | 0.977 |
| Race Black (only):Neonatal sex Female |  |  | 0.87 (0.81 - 0.93) | <0.001 | <0.001 |
| Race AIAN (only):Neonatal sex Female |  |  | 0.95 (0.78 - 1.15) | 0.594 | 0.757 |
| Race Asian (only):Neonatal sex Female |  |  | 1.00 (0.90 - 1.10) | 0.973 | 0.977 |
| Race NHOPI (only):Neonatal sex Female |  |  | 0.86 (0.58 - 1.27) | 0.450 | 0.693 |
| Race More than one Race:Neonatal sex Female |  |  | 1.02 (0.87 - 1.18) | 0.845 | 0.892 |
| Race Black (only):Birth weight (centiles) <10th centile |  |  | 1.01 (0.92 - 1.12) | 0.792 | 0.861 |
| Race AIAN (only):Birth weight (centiles) <10th centile |  |  | 0.84 (0.62 - 1.14) | 0.268 | 0.552 |
| Race Asian (only):Birth weight (centiles) <10th centile |  |  | 0.96 (0.82 - 1.12) | 0.608 | 0.757 |
| Race NHOPI (only):Birth weight (centiles) <10th centile |  |  | 1.43 (0.77 - 2.66) | 0.254 | 0.552 |
| Race More than one Race:Birth weight (centiles) <10th centile |  |  | 0.87 (0.68 - 1.11) | 0.261 | 0.552 |
| Race Black (only):Birth weight (centiles) 10-49th centile |  |  | 0.92 (0.85 - 1.00) | 0.053 | 0.182 |
| Race AIAN (only):Birth weight (centiles) 10-49th centile |  |  | 0.92 (0.73 - 1.15) | 0.453 | 0.693 |
| Race Asian (only):Birth weight (centiles) 10-49th centile |  |  | 0.94 (0.83 - 1.06) | 0.306 | 0.575 |
| Race NHOPI (only):Birth weight (centiles) 10-49th centile |  |  | 0.90 (0.57 - 1.43) | 0.655 | 0.757 |
| Race More than one Race:Birth weight (centiles) 10-49th centile |  |  | 1.05 (0.88 - 1.26) | 0.573 | 0.757 |
| Race Black (only):Birth weight (centiles) >90 centile |  |  | 1.06 (0.95 - 1.20) | 0.301 | 0.575 |
| Race AIAN (only):Birth weight (centiles) >90 centile |  |  | 1.23 (0.92 - 1.65) | 0.162 | 0.404 |
| Race Asian (only):Birth weight (centiles) >90 centile |  |  | 1.04 (0.85 - 1.28) | 0.681 | 0.769 |
| Race NHOPI (only):Birth weight (centiles) >90 centile |  |  | 0.70 (0.37 - 1.32) | 0.270 | 0.552 |
| Race More than one Race:Birth weight (centiles) >90 centile |  |  | 1.14 (0.87 - 1.50) | 0.343 | 0.609 |
| Race Black (only):Pre-pregnancy BMI (kg/m²) |  |  | 1.02 (1.01 - 1.02) | <0.001 | <0.001 |
| Race AIAN (only):Pre-pregnancy BMI (kg/m²) |  |  | 1.02 (1.00 - 1.03) | 0.022 | 0.090 |
| Race Asian (only):Pre-pregnancy BMI (kg/m²) |  |  | 1.00 (0.99 - 1.01) | 0.517 | 0.734 |
| Race NHOPI (only):Pre-pregnancy BMI (kg/m²) |  |  | 1.01 (0.99 - 1.04) | 0.378 | 0.641 |
| Race More than one Race:Pre-pregnancy BMI (kg/m²) |  |  | 1.00 (0.99 - 1.02) | 0.431 | 0.690 |
| Maternal age (years) <25 years:Neonatal sex Female |  |  | 1.13 (1.07 - 1.20) | <0.001 | <0.001 |
| Maternal age (years) 25-29 years:Neonatal sex Female |  |  | 1.02 (0.97 - 1.09) | 0.411 | 0.669 |
| Maternal age (years) 35-39 years:Neonatal sex Female |  |  | 0.96 (0.89 - 1.03) | 0.239 | 0.547 |
| Maternal age (years) ≥40 years:Neonatal sex Female |  |  | 0.93 (0.82 - 1.05) | 0.254 | 0.552 |
| Maternal age (years) <25 years:Birth weight (centiles) <10th centile |  |  | 0.94 (0.85 - 1.03) | 0.203 | 0.476 |
| Maternal age (years) 25-29 years:Birth weight (centiles) <10th centile |  |  | 0.88 (0.80 - 0.97) | 0.008 | 0.038 |
| Maternal age (years) 35-39 years:Birth weight (centiles) <10th centile |  |  | 1.03 (0.92 - 1.16) | 0.602 | 0.757 |
| Maternal age (years) ≥40 years:Birth weight (centiles) <10th centile |  |  | 1.03 (0.84 - 1.26) | 0.787 | 0.861 |
| Maternal age (years) <25 years:Birth weight (centiles) 10-49th centile |  |  | 0.95 (0.89 - 1.03) | 0.203 | 0.476 |
| Maternal age (years) 25-29 years:Birth weight (centiles) 10-49th centile |  |  | 0.96 (0.90 - 1.03) | 0.279 | 0.553 |
| Maternal age (years) 35-39 years:Birth weight (centiles) 10-49th centile |  |  | 1.05 (0.96 - 1.13) | 0.282 | 0.553 |
| Maternal age (years) ≥40 years:Birth weight (centiles) 10-49th centile |  |  | 0.96 (0.84 - 1.11) | 0.614 | 0.757 |
| Maternal age (years) <25 years:Birth weight (centiles) >90 centile |  |  | 1.09 (0.98 - 1.22) | 0.116 | 0.338 |
| Maternal age (years) 25-29 years:Birth weight (centiles) >90 centile |  |  | 0.99 (0.89 - 1.09) | 0.798 | 0.861 |
| Maternal age (years) 35-39 years:Birth weight (centiles) >90 centile |  |  | 0.95 (0.84 - 1.06) | 0.353 | 0.616 |
| Maternal age (years) ≥40 years:Birth weight (centiles) >90 centile |  |  | 0.98 (0.81 - 1.19) | 0.855 | 0.893 |
| Maternal age (years) <25 years:Pre-pregnancy BMI (kg/m²) |  |  | 1.00 (1.00 - 1.01) | 0.051 | 0.180 |
| Maternal age (years) 25-29 years:Pre-pregnancy BMI (kg/m²) |  |  | 1.00 (1.00 - 1.01) | 0.627 | 0.757 |
| Maternal age (years) 35-39 years:Pre-pregnancy BMI (kg/m²) |  |  | 1.00 (1.00 - 1.01) | 0.635 | 0.757 |
| Maternal age (years) ≥40 years:Pre-pregnancy BMI (kg/m²) |  |  | 1.01 (1.00 - 1.02) | 0.004 | 0.021 |
| Birth weight (centiles) <10th centile:Pre-pregnancy BMI (kg/m²) |  |  | 0.99 (0.98 - 0.99) | <0.001 | <0.001 |
| Birth weight (centiles) 10-49th centile:Pre-pregnancy BMI (kg/m²) |  |  | 0.99 (0.99 - 0.99) | <0.001 | <0.001 |
| Birth weight (centiles) >90 centile:Pre-pregnancy BMI (kg/m²) |  |  | 1.01 (1.00 - 1.01) | 0.071 | 0.228 |
| **E) BMI at delivery, no imputation, no interaction** |  |  |  |  |  |
| Nulliparity | 1.70 (1.66 - 1.74) | <0.001 | 2.13 (2.05 - 2.21) | <0.001 | <0.001 |
| Race |  |  |  |  |  |
| White (only) | Reference | 1.000 | Reference | 1.000 |  |
| Black (only) | 0.54 (0.53 - 0.56) | <0.001 | 0.56 (0.53 - 0.59) | <0.001 | <0.001 |
| AIAN (only) | 1.15 (1.05 - 1.27) | 0.002 | 1.19 (1.04 - 1.36) | 0.010 | 0.013 |
| Asian (only) | 1.10 (1.05 - 1.16) | <0.001 | 0.81 (0.75 - 0.87) | <0.001 | <0.001 |
| NHOPI (only) | 0.92 (0.76 - 1.12) | 0.409 | 0.90 (0.69 - 1.16) | 0.412 | 0.476 |
| More than one race | 1.13 (1.04 - 1.21) | 0.002 | 0.97 (0.88 - 1.08) | 0.611 | 0.611 |
| Maternal age (years) |  |  |  |  |  |
| <25 years | 0.70 (0.68 - 0.72) | <0.001 | 0.73 (0.69 - 0.77) | <0.001 | <0.001 |
| 25-29 years | 0.88 (0.86 - 0.91) | <0.001 | 0.92 (0.88 - 0.96) | <0.001 | <0.001 |
| 30-34 years | Reference | 1.000 | Reference | 1.000 |  |
| 35-39 years | 0.93 (0.90 - 0.96) | <0.001 | 0.90 (0.85 - 0.94) | <0.001 | <0.001 |
| ≥40 years | 0.84 (0.79 - 0.89) | <0.001 | 0.79 (0.72 - 0.86) | <0.001 | <0.001 |
| Neonatal sex |  |  |  |  |  |
| Female | 1.13 (1.10 - 1.15) | <0.001 | 1.09 (1.05 - 1.13) | <0.001 | <0.001 |
| Male | Reference | 1.000 | Reference | 1.000 |  |
| Birth weight (centiles) |  |  |  |  |  |
| <10th centile | 1.03 (1.00 - 1.07) | 0.047 | 1.10 (1.04 - 1.16) | <0.001 | 0.001 |
| 10-49th centile | 1.05 (1.03 - 1.08) | <0.001 | 1.08 (1.04 - 1.12) | <0.001 | <0.001 |
| 50-90th centile | Reference | 1.000 | Reference | 1.000 |  |
| >90 centile | 0.93 (0.89 - 0.96) | <0.001 | 0.98 (0.92 - 1.04) | 0.495 | 0.530 |
| BMI at delivery (kg/m²) | 1.01 (1.01 - 1.01) | <0.001 | 1.02 (1.02 - 1.02) | <0.001 | <0.001 |
| **F) BMI at delivery, imputation, no interaction** |  |  |  |  |  |
| Nulliparity | 1.70 (1.66 - 1.74) | <0.001 | 1.88 (1.84 - 1.93) | <0.001 | <0.001 |
| Race |  |  |  |  |  |
| White (only) | Reference | 1.000 | Reference | 1.000 |  |
| Black (only) | 0.54 (0.53 - 0.56) | <0.001 | 0.59 (0.57 - 0.61) | <0.001 | <0.001 |
| AIAN (only) | 1.15 (1.05 - 1.27) | 0.002 | 1.30 (1.18 - 1.42) | <0.001 | <0.001 |
| Asian (only) | 1.10 (1.05 - 1.16) | <0.001 | 0.99 (0.94 - 1.04) | 0.781 | 0.836 |
| NHOPI (only) | 0.92 (0.76 - 1.12) | 0.409 | 1.01 (0.84 - 1.23) | 0.901 | 0.901 |
| More than one race | 1.13 (1.04 - 1.21) | 0.002 | 1.17 (1.08 - 1.26) | <0.001 | <0.001 |
| Maternal age (years) |  |  |  |  |  |
| <25 years | 0.70 (0.68 - 0.72) | <0.001 | 0.62 (0.60 - 0.64) | <0.001 | <0.001 |
| 25-29 years | 0.88 (0.86 - 0.91) | <0.001 | 0.85 (0.83 - 0.88) | <0.001 | <0.001 |
| 30-34 years | Reference | 1.000 | Reference | 1.000 |  |
| 35-39 years | 0.93 (0.90 - 0.96) | <0.001 | 0.99 (0.96 - 1.02) | 0.525 | 0.605 |
| ≥40 years | 0.84 (0.79 - 0.89) | <0.001 | 0.93 (0.87 - 0.98) | 0.012 | 0.018 |
| Neonatal sex |  |  |  |  |  |
| Female | 1.13 (1.10 - 1.15) | <0.001 | 1.11 (1.09 - 1.14) | <0.001 | <0.001 |
| Male | Reference | 1.000 | Reference | 1.000 |  |
| Birth weight (centiles) |  |  |  |  |  |
| <10th centile | 1.03 (1.00 - 1.07) | 0.051 | 1.04 (1.01 - 1.08) | 0.020 | 0.027 |
| 10-49th centile | 1.05 (1.03 - 1.08) | <0.001 | 1.04 (1.01 - 1.06) | 0.005 | 0.009 |
| 50-90th centile | Reference | 1.000 | Reference | 1.000 |  |
| >90 centile | 0.92 (0.89 - 0.96) | <0.001 | 0.97 (0.93 - 1.00) | 0.079 | 0.098 |
| BMI at delivery (kg/m²) | 1.00 (1.00 - 1.00) | <0.001 | 1.01 (1.00 - 1.01) | <0.001 | <0.001 |
| **G) BMI at delivery, no imputation, interaction** |  |  |  |  |  |
| Nulliparity | 1.70 (1.66 - 1.74) | <0.001 | 2.75 (2.50 - 3.02) | <0.001 | <0.001 |
| Race |  |  |  |  |  |
| White (only) | Reference | 1.000 | Reference | 1.000 |  |
| Black (only) | 0.54 (0.53 - 0.56) | <0.001 | 0.66 (0.59 - 0.74) | <0.001 | <0.001 |
| AIAN (only) | 1.15 (1.05 - 1.27) | 0.002 | 1.11 (0.87 - 1.40) | 0.403 | 0.594 |
| Asian (only) | 1.10 (1.05 - 1.16) | <0.001 | 0.92 (0.79 - 1.06) | 0.245 | 0.426 |
| NHOPI (only) | 0.92 (0.76 - 1.12) | 0.409 | 0.73 (0.46 - 1.16) | 0.181 | 0.349 |
| More than one race | 1.13 (1.04 - 1.21) | 0.002 | 0.93 (0.76 - 1.13) | 0.455 | 0.602 |
| Maternal age (years) |  |  |  |  |  |
| <25 years | 0.70 (0.68 - 0.72) | <0.001 | 0.48 (0.37 - 0.62) | <0.001 | <0.001 |
| 25-29 years | 0.88 (0.86 - 0.91) | <0.001 | 0.92 (0.72 - 1.16) | 0.475 | 0.602 |
| 30-34 years | Reference | 1.000 | Reference | 1.000 |  |
| 35-39 years | 0.93 (0.90 - 0.96) | <0.001 | 0.94 (0.72 - 1.23) | 0.667 | 0.706 |
| ≥40 years | 0.84 (0.79 - 0.89) | <0.001 | 0.56 (0.35 - 0.89) | 0.014 | 0.045 |
| Neonatal sex |  |  |  |  |  |
| Female | 1.13 (1.10 - 1.15) | <0.001 | 1.04 (0.98 - 1.11) | 0.232 | 0.418 |
| Male | Reference | 1.000 | Reference | 1.000 |  |
| Birth weight (centiles) |  |  |  |  |  |
| <10th centile | 1.03 (1.00 - 1.07) | 0.047 | 1.71 (1.29 - 2.27) | <0.001 | <0.001 |
| 10-49th centile | 1.05 (1.03 - 1.08) | <0.001 | 1.53 (1.24 - 1.89) | <0.001 | <0.001 |
| 50-90th centile | Reference | 1.000 | Reference | 1.000 |  |
| >90 centile | 0.93 (0.89 - 0.96) | <0.001 | 0.73 (0.53 - 1.01) | 0.056 | 0.144 |
| BMI at delivery (kg/m²) | 1.01 (1.01 - 1.01) | <0.001 | 1.02 (1.01 - 1.03) | <0.001 | <0.001 |
| Nulliparity:Race Black (only) |  |  | 0.69 (0.61 - 0.78) | <0.001 | <0.001 |
| Nulliparity:Race AIAN (only) |  |  | 1.09 (0.79 - 1.51) | 0.584 | 0.652 |
| Nulliparity:Race Asian (only) |  |  | 0.78 (0.67 - 0.91) | 0.001 | 0.005 |
| Nulliparity:Race NHOPI (only) |  |  | 2.28 (1.16 - 4.46) | 0.017 | 0.050 |
| Nulliparity:Race More than one Race |  |  | 0.92 (0.73 - 1.15) | 0.468 | 0.602 |
| Nulliparity:Maternal age (years) <25 years |  |  | 0.68 (0.62 - 0.76) | <0.001 | <0.001 |
| Nulliparity:Maternal age (years) 25-29 years |  |  | 0.86 (0.78 - 0.95) | 0.002 | 0.009 |
| Nulliparity:Maternal age (years) 35-39 years |  |  | 0.94 (0.83 - 1.06) | 0.312 | 0.496 |
| Nulliparity:Maternal age (years) ≥40 years |  |  | 0.80 (0.62 - 1.04) | 0.100 | 0.225 |
| Nulliparity:Neonatal sex Female |  |  | 1.11 (1.03 - 1.20) | 0.007 | 0.023 |
| Nulliparity:Birth weight (centiles) <10th centile |  |  | 0.67 (0.60 - 0.75) | <0.001 | <0.001 |
| Nulliparity:Birth weight (centiles) 10-49th centile |  |  | 0.87 (0.80 - 0.95) | 0.002 | 0.009 |
| Nulliparity:Birth weight (centiles) >90 centile |  |  | 1.07 (0.92 - 1.23) | 0.384 | 0.592 |
| Race Black (only):Birth weight (centiles) <10th centile |  |  | 0.92 (0.77 - 1.08) | 0.308 | 0.496 |
| Race AIAN (only):Birth weight (centiles) <10th centile |  |  | 1.35 (0.86 - 2.09) | 0.188 | 0.351 |
| Race Asian (only):Birth weight (centiles) <10th centile |  |  | 0.93 (0.75 - 1.16) | 0.523 | 0.615 |
| Race NHOPI (only):Birth weight (centiles) <10th centile |  |  | 1.51 (0.68 - 3.36) | 0.311 | 0.496 |
| Race More than one Race:Birth weight (centiles) <10th centile |  |  | 0.91 (0.66 - 1.27) | 0.592 | 0.652 |
| Race Black (only):Birth weight (centiles) 10-49th centile |  |  | 0.90 (0.79 - 1.03) | 0.142 | 0.294 |
| Race AIAN (only):Birth weight (centiles) 10-49th centile |  |  | 0.91 (0.67 - 1.25) | 0.573 | 0.652 |
| Race Asian (only):Birth weight (centiles) 10-49th centile |  |  | 0.93 (0.78 - 1.10) | 0.407 | 0.594 |
| Race NHOPI (only):Birth weight (centiles) 10-49th centile |  |  | 1.00 (0.52 - 1.92) | 0.998 | 0.998 |
| Race More than one Race:Birth weight (centiles) 10-49th centile |  |  | 1.10 (0.86 - 1.41) | 0.438 | 0.602 |
| Race Black (only):Birth weight (centiles) >90 centile |  |  | 1.15 (0.94 - 1.40) | 0.169 | 0.337 |
| Race AIAN (only):Birth weight (centiles) >90 centile |  |  | 1.39 (0.94 - 2.06) | 0.097 | 0.225 |
| Race Asian (only):Birth weight (centiles) >90 centile |  |  | 1.10 (0.82 - 1.49) | 0.523 | 0.615 |
| Race NHOPI (only):Birth weight (centiles) >90 centile |  |  | 0.74 (0.32 - 1.71) | 0.479 | 0.602 |
| Race More than one Race:Birth weight (centiles) >90 centile |  |  | 1.54 (1.04 - 2.27) | 0.031 | 0.089 |
| Maternal age (years) <25 years:Neonatal sex Female |  |  | 1.16 (1.05 - 1.28) | 0.004 | 0.014 |
| Maternal age (years) 25-29 years:Neonatal sex Female |  |  | 0.97 (0.89 - 1.06) | 0.521 | 0.615 |
| Maternal age (years) 35-39 years:Neonatal sex Female |  |  | 0.96 (0.87 - 1.07) | 0.473 | 0.602 |
| Maternal age (years) ≥40 years:Neonatal sex Female |  |  | 1.00 (0.84 - 1.19) | 0.987 | 0.998 |
| Maternal age (years) <25 years:BMI at delivery (kg/m²) |  |  | 1.02 (1.01 - 1.02) | <0.001 | <0.001 |
| Maternal age (years) 25-29 years:BMI at delivery (kg/m²) |  |  | 1.00 (0.99 - 1.01) | 0.614 | 0.663 |
| Maternal age (years) 35-39 years:BMI at delivery (kg/m²) |  |  | 1.00 (0.99 - 1.01) | 0.995 | 0.998 |
| Maternal age (years) ≥40 years:BMI at delivery (kg/m²) |  |  | 1.01 (1.00 - 1.03) | 0.077 | 0.188 |
| Birth weight (centiles) <10th centile:BMI at delivery (kg/m²) |  |  | 0.99 (0.98 - 1.00) | 0.037 | 0.099 |
| Birth weight (centiles) 10-49th centile:BMI at delivery (kg/m²) |  |  | 0.99 (0.98 - 1.00) | 0.004 | 0.014 |
| Birth weight (centiles) >90 centile:BMI at delivery (kg/m²) |  |  | 1.01 (1.00 - 1.02) | 0.127 | 0.275 |
| **H) BMI at delivery, imputation, interaction** |  |  |  |  |  |
| Nulliparity | 1.70 (1.66 - 1.74) | <0.001 | 2.56 (2.42 - 2.72) | <0.001 | <0.001 |
| Race |  |  |  |  |  |
| White (only) | Reference | 1.000 | Reference | 1.000 |  |
| Black (only) | 0.54 (0.53 - 0.56) | <0.001 | 0.51 (0.42 - 0.62) | <0.001 | <0.001 |
| AIAN (only) | 1.15 (1.05 - 1.27) | 0.002 | 0.75 (0.43 - 1.30) | 0.302 | 0.562 |
| Asian (only) | 1.10 (1.05 - 1.16) | <0.001 | 1.09 (0.82 - 1.46) | 0.553 | 0.762 |
| NHOPI (only) | 0.92 (0.76 - 1.12) | 0.409 | 0.50 (0.15 - 1.62) | 0.246 | 0.543 |
| More than one race | 1.13 (1.04 - 1.21) | 0.002 | 1.32 (0.86 - 2.02) | 0.210 | 0.531 |
| Maternal age (years) |  |  |  |  |  |
| <25 years | 0.70 (0.68 - 0.72) | <0.001 | 0.72 (0.67 - 0.77) | <0.001 | <0.001 |
| 25-29 years | 0.88 (0.86 - 0.91) | <0.001 | 0.89 (0.84 - 0.95) | <0.001 | 0.001 |
| 30-34 years | Reference | 1.000 | Reference | 1.000 |  |
| 35-39 years | 0.93 (0.90 - 0.96) | <0.001 | 1.03 (0.96 - 1.11) | 0.403 | 0.643 |
| ≥40 years | 0.84 (0.79 - 0.89) | <0.001 | 1.00 (0.88 - 1.13) | 0.958 | 0.989 |
| Neonatal sex |  |  |  |  |  |
| Female | 1.13 (1.10 - 1.15) | <0.001 | 1.10 (1.05 - 1.15) | <0.001 | <0.001 |
| Male | Reference | 1.000 | Reference | 1.000 |  |
| Birth weight (centiles) |  |  |  |  |  |
| <10th centile | 1.03 (1.00 - 1.07) | 0.051 | 2.08 (1.73 - 2.50) | <0.001 | <0.001 |
| 10-49th centile | 1.05 (1.03 - 1.08) | <0.001 | 1.56 (1.36 - 1.79) | <0.001 | <0.001 |
| 50-90th centile | Reference | 1.000 | Reference | 1.000 |  |
| >90 centile | 0.92 (0.89 - 0.96) | <0.001 | 0.76 (0.62 - 0.92) | 0.006 | 0.027 |
| BMI at delivery (kg/m²) | 1.00 (1.00 - 1.00) | <0.001 | 1.01 (1.01 - 1.01) | <0.001 | <0.001 |
| Nulliparity:Race Black (only) |  |  | 0.80 (0.74 - 0.86) | <0.001 | <0.001 |
| Nulliparity:Race AIAN (only) |  |  | 1.14 (0.90 - 1.45) | 0.272 | 0.550 |
| Nulliparity:Race Asian (only) |  |  | 0.88 (0.79 - 0.97) | 0.014 | 0.057 |
| Nulliparity:Race NHOPI (only) |  |  | 1.54 (0.93 - 2.54) | 0.092 | 0.289 |
| Nulliparity:Race More than one Race |  |  | 0.91 (0.77 - 1.07) | 0.259 | 0.548 |
| Nulliparity:Maternal age (years) <25 years |  |  | 0.66 (0.62 - 0.70) | <0.001 | <0.001 |
| Nulliparity:Maternal age (years) 25-29 years |  |  | 0.87 (0.82 - 0.93) | <0.001 | <0.001 |
| Nulliparity:Maternal age (years) 35-39 years |  |  | 0.92 (0.84 - 1.00) | 0.046 | 0.157 |
| Nulliparity:Maternal age (years) ≥40 years |  |  | 0.84 (0.71 - 1.00) | 0.044 | 0.153 |
| Nulliparity:Birth weight (centiles) <10th centile |  |  | 0.72 (0.67 - 0.77) | <0.001 | <0.001 |
| Nulliparity:Birth weight (centiles) 10-49th centile |  |  | 0.90 (0.85 - 0.95) | <0.001 | 0.001 |
| Nulliparity:Birth weight (centiles) >90 centile |  |  | 0.96 (0.88 - 1.05) | 0.357 | 0.601 |
| Race Black (only):Maternal age (years) <25 years |  |  | 1.01 (0.92 - 1.11) | 0.840 | 0.917 |
| Race AIAN (only):Maternal age (years) <25 years |  |  | 0.95 (0.72 - 1.25) | 0.717 | 0.858 |
| Race Asian (only):Maternal age (years) <25 years |  |  | 1.22 (1.02 - 1.47) | 0.030 | 0.108 |
| Race NHOPI (only):Maternal age (years) <25 years |  |  | 1.25 (0.69 - 2.26) | 0.466 | 0.707 |
| Race More than one Race:Maternal age (years) <25 years |  |  | 0.97 (0.79 - 1.20) | 0.801 | 0.900 |
| Race Black (only):Maternal age (years) 25-29 years |  |  | 1.01 (0.92 - 1.12) | 0.783 | 0.891 |
| Race AIAN (only):Maternal age (years) 25-29 years |  |  | 1.10 (0.84 - 1.45) | 0.490 | 0.730 |
| Race Asian (only):Maternal age (years) 25-29 years |  |  | 1.11 (0.97 - 1.26) | 0.123 | 0.361 |
| Race NHOPI (only):Maternal age (years) 25-29 years |  |  | 1.17 (0.67 - 2.05) | 0.577 | 0.772 |
| Race More than one Race:Maternal age (years) 25-29 years |  |  | 0.95 (0.76 - 1.17) | 0.614 | 0.798 |
| Race Black (only):Maternal age (years) 35-39 years |  |  | 1.04 (0.92 - 1.17) | 0.534 | 0.748 |
| Race AIAN (only):Maternal age (years) 35-39 years |  |  | 0.89 (0.64 - 1.25) | 0.512 | 0.739 |
| Race Asian (only):Maternal age (years) 35-39 years |  |  | 1.03 (0.90 - 1.17) | 0.705 | 0.856 |
| Race NHOPI (only):Maternal age (years) 35-39 years |  |  | 0.99 (0.50 - 1.95) | 0.972 | 0.989 |
| Race More than one Race:Maternal age (years) 35-39 years |  |  | 0.82 (0.63 - 1.07) | 0.145 | 0.414 |
| Race Black (only):Maternal age (years) ≥40 years |  |  | 1.19 (0.98 - 1.44) | 0.085 | 0.277 |
| Race AIAN (only):Maternal age (years) ≥40 years |  |  | 0.70 (0.36 - 1.37) | 0.292 | 0.562 |
| Race Asian (only):Maternal age (years) ≥40 years |  |  | 1.12 (0.90 - 1.40) | 0.320 | 0.562 |
| Race NHOPI (only):Maternal age (years) ≥40 years |  |  | 0.73 (0.20 - 2.74) | 0.644 | 0.800 |
| Race More than one Race:Maternal age (years) ≥40 years |  |  | 1.01 (0.66 - 1.56) | 0.955 | 0.989 |
| Race Black (only):Neonatal sex Female |  |  | 0.87 (0.82 - 0.93) | <0.001 | <0.001 |
| Race AIAN (only):Neonatal sex Female |  |  | 0.95 (0.79 - 1.16) | 0.634 | 0.800 |
| Race Asian (only):Neonatal sex Female |  |  | 1.00 (0.91 - 1.11) | 0.985 | 0.989 |
| Race NHOPI (only):Neonatal sex Female |  |  | 0.87 (0.59 - 1.30) | 0.498 | 0.732 |
| Race More than one Race:Neonatal sex Female |  |  | 1.02 (0.87 - 1.18) | 0.846 | 0.917 |
| Race Black (only):Birth weight (centiles) <10th centile |  |  | 1.00 (0.90 - 1.11) | 0.989 | 0.989 |
| Race AIAN (only):Birth weight (centiles) <10th centile |  |  | 0.84 (0.62 - 1.14) | 0.251 | 0.543 |
| Race Asian (only):Birth weight (centiles) <10th centile |  |  | 0.97 (0.83 - 1.12) | 0.651 | 0.800 |
| Race NHOPI (only):Birth weight (centiles) <10th centile |  |  | 1.45 (0.78 - 2.70) | 0.235 | 0.543 |
| Race More than one Race:Birth weight (centiles) <10th centile |  |  | 0.86 (0.67 - 1.10) | 0.222 | 0.543 |
| Race Black (only):Birth weight (centiles) 10-49th centile |  |  | 0.91 (0.84 - 0.99) | 0.023 | 0.086 |
| Race AIAN (only):Birth weight (centiles) 10-49th centile |  |  | 0.90 (0.72 - 1.13) | 0.370 | 0.608 |
| Race Asian (only):Birth weight (centiles) 10-49th centile |  |  | 0.95 (0.84 - 1.07) | 0.374 | 0.608 |
| Race NHOPI (only):Birth weight (centiles) 10-49th centile |  |  | 0.93 (0.58 - 1.47) | 0.745 | 0.858 |
| Race More than one Race:Birth weight (centiles) 10-49th centile |  |  | 1.05 (0.88 - 1.26) | 0.599 | 0.790 |
| Race Black (only):Birth weight (centiles) >90 centile |  |  | 1.07 (0.95 - 1.21) | 0.242 | 0.543 |
| Race AIAN (only):Birth weight (centiles) >90 centile |  |  | 1.23 (0.92 - 1.64) | 0.170 | 0.442 |
| Race Asian (only):Birth weight (centiles) >90 centile |  |  | 1.04 (0.84 - 1.28) | 0.728 | 0.858 |
| Race NHOPI (only):Birth weight (centiles) >90 centile |  |  | 0.73 (0.39 - 1.37) | 0.327 | 0.562 |
| Race More than one Race:Birth weight (centiles) >90 centile |  |  | 1.15 (0.87 - 1.51) | 0.323 | 0.562 |
| Race Black (only):BMI at delivery (kg/m²) |  |  | 1.01 (1.00 - 1.01) | <0.001 | <0.001 |
| Race AIAN (only):BMI at delivery (kg/m²) |  |  | 1.02 (1.00 - 1.03) | 0.015 | 0.059 |
| Race Asian (only):BMI at delivery (kg/m²) |  |  | 1.00 (0.99 - 1.01) | 0.521 | 0.741 |
| Race NHOPI (only):BMI at delivery (kg/m²) |  |  | 1.02 (0.99 - 1.05) | 0.233 | 0.543 |
| Race More than one Race:BMI at delivery (kg/m²) |  |  | 1.00 (0.99 - 1.01) | 0.738 | 0.858 |
| Maternal age (years) <25 years:Neonatal sex Female |  |  | 1.13 (1.07 - 1.20) | <0.001 | <0.001 |
| Maternal age (years) 25-29 years:Neonatal sex Female |  |  | 1.02 (0.97 - 1.09) | 0.426 | 0.657 |
| Maternal age (years) 35-39 years:Neonatal sex Female |  |  | 0.96 (0.90 - 1.03) | 0.266 | 0.550 |
| Maternal age (years) ≥40 years:Neonatal sex Female |  |  | 0.94 (0.83 - 1.06) | 0.290 | 0.562 |
| Maternal age (years) <25 years:Birth weight (centiles) <10th centile |  |  | 0.93 (0.85 - 1.03) | 0.156 | 0.430 |
| Maternal age (years) 25-29 years:Birth weight (centiles) <10th centile |  |  | 0.88 (0.80 - 0.97) | 0.009 | 0.039 |
| Maternal age (years) 35-39 years:Birth weight (centiles) <10th centile |  |  | 1.03 (0.92 - 1.16) | 0.635 | 0.800 |
| Maternal age (years) ≥40 years:Birth weight (centiles) <10th centile |  |  | 1.02 (0.83 - 1.24) | 0.879 | 0.942 |
| Maternal age (years) <25 years:Birth weight (centiles) 10-49th centile |  |  | 0.95 (0.88 - 1.02) | 0.165 | 0.441 |
| Maternal age (years) 25-29 years:Birth weight (centiles) 10-49th centile |  |  | 0.97 (0.90 - 1.03) | 0.320 | 0.562 |
| Maternal age (years) 35-39 years:Birth weight (centiles) 10-49th centile |  |  | 1.04 (0.96 - 1.13) | 0.312 | 0.562 |
| Maternal age (years) ≥40 years:Birth weight (centiles) 10-49th centile |  |  | 0.96 (0.83 - 1.11) | 0.573 | 0.772 |
| Maternal age (years) <25 years:Birth weight (centiles) >90 centile |  |  | 1.09 (0.98 - 1.22) | 0.113 | 0.343 |
| Maternal age (years) 25-29 years:Birth weight (centiles) >90 centile |  |  | 0.99 (0.89 - 1.09) | 0.833 | 0.917 |
| Maternal age (years) 35-39 years:Birth weight (centiles) >90 centile |  |  | 0.95 (0.85 - 1.07) | 0.413 | 0.647 |
| Maternal age (years) ≥40 years:Birth weight (centiles) >90 centile |  |  | 1.00 (0.83 - 1.22) | 0.983 | 0.989 |
| Birth weight (centiles) <10th centile:BMI at delivery (kg/m²) |  |  | 0.98 (0.98 - 0.99) | <0.001 | <0.001 |
| Birth weight (centiles) 10-49th centile:BMI at delivery (kg/m²) |  |  | 0.99 (0.99 - 0.99) | <0.001 | <0.001 |
| Birth weight (centiles) >90 centile:BMI at delivery (kg/m²) |  |  | 1.01 (1.00 - 1.01) | 0.011 | 0.047 |

Acronyms: AIAN = American Indian and Alaska Native; BMI = body mass index; ECV = external cephalic version; NHOPI = Native Hawaiian and Other Pacific Islanders.
